# Nano-mediated delivery of double-stranded RNA for gene therapy of glioblastoma multiforme

**DOI:** 10.1371/journal.pone.0213852

**Published:** 2019-03-19

**Authors:** Małgorzata Grabowska, Bartosz F. Grześkowiak, Kosma Szutkowski, Dariusz Wawrzyniak, Paweł Głodowicz, Jan Barciszewski, Stefan Jurga, Katarzyna Rolle, Radosław Mrówczyński

**Affiliations:** 1 Department of Molecular Neurooncology, Institute of Bioorganic Chemistry Polish Academy of Science, Poznan, Poland; 2 NanoBioMedical Centre, Adam Mickiewicz University in Poznan, Poznan, Poland; 3 Department of Epigenetics, Institute of Bioorganic Chemistry Polish Academy of Science, Poznan, Poland; 4 Centre for Advanced Technologies, Poznan, Poland; Sechenov First Medical University, RUSSIAN FEDERATION

## Abstract

Glioblastoma multiforme (GBM) is the most common type of malignant gliomas, characterized by genetic instability, intratumoral histopathological variability and unpredictable clinical behavior. Disappointing results in the treatment of gliomas with surgery, radiation and chemotherapy have fueled a search for new therapeutic targets and treatment modalities. Here we report new approach towards RNA interference therapy of glioblastoma multiforme based on the magnetic nanoparticles delivery of the double-stranded RNA (dsRNA) with homological sequences to mRNA of tenascin-C (TN-C), named ATN-RNA. The obtained nanocomposite consisted of polyethyleneimine (PEI) coated magnetic nanoparticles conjugated to the dsRNA show high efficiency in ATN-RNA delivery, resulting not only in significant TN-C expression level suppressesion, but also impairing the tumor cells migration. Moreover, synthesized nanomaterials show high contrast properties in magnetic resonance imaging (MRI) and low cytotoxicity combining with lack of induction of interferon response. We believe that the present work is a successful combination of effective, functional, non-immunostimulatory dsRNA delivery system based on magnetic nanoparticles with high potential for further application in GBM therapy.

## Introduction

Although accounting for less than 2% of adult cancers, gliomas are the most common form of malignant primary brain tumor in adults.[1] Glioblastoma multiforme constitutes 25% of all malignant nervous system tumors and the median overall survival remains around 12–15 months, even after combination treatments of cytoreductive surgical resection, radiotherapy, and adjuvant oral chemotherapy with temozolomide[2,3,4,5] The recent medical treatment strategies have been progressing toward individualized therapy and many targeted drugs have been investigated, but the identification of molecular biomarkers in GBM as well as the novel drugs delivery strategies are still of considerable therapeutic importance.

Among recently developed new methods towards GBM treatment, a lot of attention has been drawn to gene therapy.[6,7,8] One of the strategy is RNA interference (RNAi), a general term referring to post-transcriptional gene silencing mediated by either degradation or translation arrest of target RNA. RNAi is triggered by gene silencing mechanism that is initiated with the introduction of dsRNA into a cell.[9] Specifically designed RNAi molecules can target mRNAs and initiate their degradation. We have previously reported that dsRNA synthesized by *in vitro* method can cleave the targeted mRNA and silenced the gene of interest expression.[10,11] We used double-stranded 164 nucleotides long double stranded RNA specific for tenascin-C (TN-C) mRNA. That agent, called ATN-RNA, induces RNAi pathway to degradation of TN-C, the extracellular matrix (ECM) protein which is highly overexpressed in brain tumor tissue. The technology was coined interference RNA intervention (iRNAi). With the approach ATN-RNA was administrated locally into the tumor’s cavity during standard neurosurgical procedures. Although the obtained clinical outcome from the experimental therapy has been found as the very promising, resulting with the improving of the quality of patients life, as well as the increasing of the overall survival, we are still far from the conclusion that the used approach can be the most effective GBM treatment. Lack of an effective delivery method for dsRNA and the instability of the nucleic acids during and/or after the delivery are still the major difficulties in gene silencing studies.

In the last decade, nanotechnology has been widely applied in synthesis of nanoscale materials with main focus on cancer therapy.[12,13,14] Especially a lot of work has been put on the development of new, multifunctional delivery systems which could overcome drawbacks of conventional treatment methods and improve existing therapies by selective enrichment of active substances in diseased tissue structures, an increase in bioavailability, the decrease of the active substance degradation and, above all, the reduction and/or avoidance of unwanted side effects.[15,16] Magnetic nanoparticles, mostly based on magnetite (Mag) have drawn so much considerable attention in the field of smart materials due to their unique advantages over other materials.[17,18] They are easy to produce and the synthesis cost is relatively small in comparison to other nanomaterials.[19] Moreover, they are physically and chemically stable, biocompatible and environmentally friendly. Therefore, they have been frequently used in gene therapy as non-viral gene carriers.[20] Another important point in the application of magnetic nanoparticles is the fact that they can be employed as contrast agents in MRI (Magnetic Resonance Imaging) what makes them a very powerful diagnostic tool.[21] Indeed, the magnetic nanoparticles have been successfully applied also in MRI of a brain tumor as well as in magnetic hyperthermia resulting in reducing of brain tumor mass.[22,23] Recently, they have been also used in gene therapy for delivery of siRNA against survivin but reported results showed moderate mortality of the cells.[24]

In the paper, we demonstrate application of magnetic nanoparticles as a multifunctional carrier for a double-stranded RNA with contrast properties in MRI. We used a previously reported ATN-RNA sequence shown to significantly reduce the expression level of TN-C. The conjugation of nanoparticles with ATN-RNA showed the more effective TN-C downregulation followed by the substantial impairment of the migration properties of cancer cells regarding the routinely used transfection agent. Furthermore, obtained nanocomposites and their complexes with ATN-RNA did not show the toxic properties and consequently, they do not stimulate the interferon induction and innate immune response. Therefore, our results shed new light on application of multifunctional magnetic nanoparticles in gene therapy of GBM utilizing RNAi approach and point promising candidate for further studies.

## Materials and methods

All reagents and solvents were of reagent-grade quality. For all experiments, Milli-Q deionized water (resistivity 18 MΩ×cm^**−1**^) was used.

### Synthesis and labeling of Mag@PEI nanoparticles

Magnetic nanoparticles coated with PEI (Mag@PEI) were synthesized according to a previously reported protocol with small modifications. Briefly, FeCl_**3**_·6H_**2**_O (Sigma-Aldrich) (135 mg, 0.5 mmol) were mixed with FeCl_**2**_·4H_**2**_O (Sigma-Aldrich) (50 mg, 0.25 mmol) in water (5 mL) and degassed with N_**2**_. The mixture was heated up to 80°C followed by addition of 1 mL of working solution (0.5 g of 25-kDa branched polyethyleneimine (PEI-25_**Br**_), 250 μL of Capstone FS-65 fluorosurfactant (Du Pont) and 2.5 mL of NH_**4**_OH dissolved in 10 mL of Milli-Q water) and constant heating was maintained for 120 min. After cooling down the mixture of nanoparticles was washed with water (2 x 150 mL) and collected by an external magnet. Finally, the NPs were redispersed in 10 mL of water. The Mag@PEI concentration was determined spectrophotometrically in terms of the iron content in an aqueous suspension of the stock nanomaterial by complexation with 1,10-phenanthroline (Sigma-Aldrich) as described previously.[25]

Nanoparticles conjugated with fluorescent dye ATTO 550 were prepared as described elsewhere [26] Briefly, 2 mL of Mag@PEI nanoparticle suspension in water (2.25 mg Fe/mL) was mixed with 490 μL 0.1 M borate buffer, pH 8.5, and 10 μL solution of ATTO 550 NHS-ester (Sigma-Aldrich) in DMSO (10 mg/mL). The resulting suspension was incubated o/n at room temperature and dialyzed against water using Slide-A-Lyzer Dialysis Cassette G2 (Thermo Fisher Scientific) with a cut-off at 3500 MW.

### Physical characterization

For transmission electron microscopy (TEM) a small amount of the sample was placed on a copper measuring grid (Formvar/Carbon 200 Mesh made by TedPella (USA)) after 5 minutes of sonication in deionized water. Then, the sample was dried in a vacuum desiccator for 24 hours. TEM images were recorded on a JEM-1400 microscope made by JEOL (Japan) at an accelerating voltage of 120 kV. The ImageJ software (Bethesda, MD, USA) was used to process TEM micrographs in order to analyze the size of nanoparticles. Magnetic measurements were performed on SQUID magnetometer at 5 K and 300 K. FT-IR spectra were recorded on Bruker Tensor 27 spectrometer in KBr pallets. Multiple light scattering (MLS) measurements were performed on Turbiscan Lab produced by Formulaction SA in 10 mL vials. Zeta (ζ) potential measurements were carried out using Zetasizer Nano-ZS ZEN 3600 produced by Malvern Instruments Ltd (UK). The experimental setup for magnetic resonance imaging studies consisted of an Agilent 9.4 T MRI preclinical scanner equipped with a 120 mm gradient coil (2 mT/m/A) and 30 mm millipede coil. In order to perform a quantitative T_**2**_ relaxation experiment (at 18°C) we employed MEMS protocol. Each echo is acquired after an excitation pulse with an increasing echo time. The parameters of the data acquisition were as follows: field of view 15 mm (FOV), matrix size 128 × 128, Gaussian-shaped pulse with 2048 μs length. The echo time was set to 4 and 10 ms in two separate experiments. In total, 16 consecutive images with varied echo time were acquired for each sample. The initial analysis was performed in VnmrJ 4.2 revision software (Santa Clara, CA, USA). The raw relaxation data points were collected from an average intensity obtained from circular Region of Interests (ROIs) and then analyzed using Origin 8.5 software (Origin Lab, Northampton, MA, USA) using simple single exponential decay function. In order to prepare MRI agarose-based phantom, nanoparticles were suspended in a hot 2% agarose solution. The hot solution was finally transferred to 10 mm plastic vials and left for full agarose gelation.

### ATN-RNA preparation

ATN-RNA was synthesized *in vitro* as it has been already published.[11] ATN DNA harboring plasmid was cleaved by EcoRI and HindIII restriction enzymes (Promega). The two strands of RNA were transcribed separately with T3 and T7 RNA polymerases from MEGAscript Transcription Kits (Ambion). Hybridization of RNA was performed in a buffer containing 20 mM Tris-HCl, pH 7.5 and 50 mM NaCl. The reaction was carried out for 3 min at 95°C, 30 min at 75°C and at the end slowly cooled down to the room temperature.

Sequence of ATN-RNA (US Patent US 8,946.400 B2)

5’ CAAGCGACAGAGUGGGGUGAACGCCACCCUGCCAGAAGAGAACCAGCCAGUGGUGUUUAACCACGUUUACAACAUCAAGCUGCCAGUGGGAUCCCAGUGUUCGGUGGAUCUGGAGUCAGCCAGUCCCCUCUUCUGGACCGGGCGGAAGUCUCGGGCGCU 3’

3’ GUUCGCUGUCUCACCCCACUUGCGGUGGGACGGUCUUCUCUUGGUCGGUCACCACAAAUUGGUGCAAAUGUUGUAGUUCGACGGUCACCCUAGGGUCACAAGCCACCUAGACCUCAGUCGGUCACCCCUCUUCUGGACCGGGCGGAAGUCUCGGGCGCU 5’

### Preparation of Mag@PEI/ATN-RNA complexes

The binding ability of ATN-RNA to Mag@PEI was performed by the gel retardation assay and UV-Vis spectrophotometry. To prepare Mag@PEI/ATN-RNA complexes, 1 μg of ATN-RNA was mixed with Mag@PEI with a series of Fe weight ratios (1, 2, 3, 4, 5, 8, 10 Mag@PEI to ATN-RNA wt:wt ratio) in the nuclease-free water, and incubated for 30 min at RT, allowing for sufficient binding of dsRNA molecules with the Mag@PEI. The Mag@PEI/ATN-RNA complexes were loaded onto 1% agarose gel for electrophoresis in TAE buffer at a constant voltage of 70 V for 30 min to visualize the ATN-RNA bands using a Digital Imaging and Analysis System II (Serva). SimplySafe fluorescent stain was used for detecting RNA in agarose gel. Meanwhile, the formed Mag@PEI/RNA complexes suspension was centrifuged (10000 rpm, 10 min) and the prepared supernatant was analyzed spectrophotometrically (NanoDrop 2000, Thermo Scientific) at A260 nm to reveal ATN-RNA content.

### Cell culture

The study was performed on a human U-118 MG cell line (ATTC) derived from a glioblastoma multiforme. Adherent cell growth was carried out in a Dulbecco's Modified Eagle Medium (DMEM, Gibco) High Glucose supplemented with 10% fetal bovine serum (FBS, Sigma-Aldrich) and 1% penicillin-streptomycin antibiotic (Sigma-Aldrich). Cells were grown at 37°C in a 95% humidified chamber with 5% CO_**2**_.

### Transfection of GBM cells

ATN-RNA transfection was performed with Mag@PEI or Lipofectamine (Invitrogen) as a carrier. The procedure was carried out on the day-old cell culture seeded on 12 or 96-well plates in the supplemented medium. In a case of the 12-well plate, 150,000 cells were seeded in 1 mL of medium, for 96-well it was 10,000 cells in 200 μL. Once the cells have reached 75–90% confluency, the medium was removed, cells were washed with phosphate-buffered saline (PBS, Sigma-Aldrich) buffer and an unsupplemented culture medium in the amount reduced by the volume of the transfection mixture was added. Transfection complexes containing Mag@PEI nanoparticles were prepared by mixing Mag@PEI suspension (100 μg Fe/mL) with ATN-RNA solution (100 ng/μL) in serum and supplement-free DMEM with an iron-to-RNA ratio of 3:1 (w/w). The mixture was further incubated at RT for 30 min to allow the components to assemble. After this time, the volumes of the prepared complexes corresponding to the proper ATN-RNA final concentration (10, 25, 50 and 100 nM) were transferred to the wells.

Lipofection was performed according to the manufacturer's recommendations. Two separate mixtures, OptiMEM (Gibco) with Lipofectamine and OptiMEM with ATN-RNA, were incubated for 5 min at the room temperature. After that, both solutions were combined. The prepared reaction mixture was incubated 20 min at the room temperature and then transferred to the appropriate wells. In addition, cells treated with Lipofectamine or Mag@PEI carriers only were used as controls.

### Cellular uptake of transfection complexes

To track the magnetic complexes in the cell, ATN-RNA was fluorescently labelled with fluorescein, according to the manufacturer’s protocol (Label IT Tracker Intracellular Nucleic Acid Localization Kits, Mirus Bio, USA). The final concentration of the fluorescein containing RNA was quantified on a spectrophotometer (NanoDrop 2000). The fluorescently labelled Mag@PEI nanoparticles were then used to assemble complexes with fluorescently labelled ATN-RNA, as described in the previous section. U-118 cells (2.5 x 10^**4**^ cells/well) were plated onto an 8-well Nunc Lab-Tek Chamber Slide (Thermo Fisher Scientific) and cultured for 24 h. Next, 50 μL of Mag@PEI/ATN-complexes (100 nM) was added and incubated with the cells at 37°C for 24 h.

For visualization under a confocal microscope, the cells were fixed with 4% formaldehyde (Sigma-Aldrich) in phosphate-buffered saline (PBS) for 15 min. The cell membranes were stained with ConcanavalinA-FITC (Life Technologies) at a concentration of 50 μg/mL, the F-actin fibers were stained with Oregon Green 488 phalloidin (Molecular Probes) at a concentration of 165 nM and the cell nuclei were stained with Hoechst 33342 (Molecular Probes) at a concentration of 8 μM. Cells were imaged using a confocal laser scanning microscope (Olympus FV1000, Japan). Image acquisition and analysis were performed with a 60x objective, a 1.4 oil immersion lens and FV10-ASW software (Olympus). Images of the Mag@PEI were visualized using 559 nm excitation and 570–590 nm emission filters, whereas ATN-RNA was visualized using 488 nm excitation and 495–545 nm emission filters. To visualize the cell membranes or cytoskeleton, 488 nm excitation and 495–545 nm emission filters were applied. The Hoechst fluorescence was detected using 405 nm excitation source and 425–475 nm emission filters. The 3D-scan of the sample was performed using Z-stack mode measurements and analyzed with Imaris software (Bitplane).

### Cytotoxicity assays

The WST-1 cell proliferation assay, as well as fluorescent cell viability assay, were carried out to assess the cytotoxicity of the nanoparticles and complexes with ATN-RNA.

In WST-1 assay (Takara), U-118 cells were seeded at the density of 10,000 cells per well in the 96-well plate. After 24 hours, medium containing an increasing concentration of tested Mag@PEI/ATN-RNA complexes (in terms of RNA concentration) was added to each well and the cells were incubated for 24 h. After incubation, 10 μL of the WST-1 Cell Proliferation Reagent was added to each well and incubated for 4 hours. After this time, the absorbance at 450 nm (reference wavelength 620 nm) was recorded against the background control, using a multiwell plate reader (Zenyth, Biochrom) and the cell viability was expressed as the respiration activity normalized to untreated cells.

In Live/Dead assay, the U-118 cells were seeded in black polystyrene 96-wells flat bottom plate with the transparent bottom (Greiner Bio-One GmbH) at densities of 10 000 cells per well. After 24 h, medium containing an increasing concentration of tested Mag@PEI/ATN-RNA complexes (in terms of RNA concentration) was added to each well. Following 24 h exposure to the complexes, cells were incubated with 2 μM calcein AM (Thermo Fisher Scientific), 2 μM ethidium homodimer-1 (Thermo Fisher Scientific) and 8 μM Hoechst 33342 containing PBS (100 μL/well) during 30 minutes at 37°C. Finally, the cells were analyzed with the IN Cell Analyzer 2000 (GE Healthcare Life Sciences, UK). Viable cells were imaged using the FITC/FITC excitation/emission filters while for the dead cells the TexasRed/TexasRed ex/em filter combination was applied. DAPI/DAPI was applied to detect the Hoechst 33342 blue signal. A minimum of 20 fields was imaged per well with a 20x magnification. Analysis of the collected images was performed with the IN Cell Developer Toolbox software (GE Healthcare Life Sciences, UK) using in-house developed protocol. First, the total cell number was retrieved from the DAPI images by means of defining and counting the nuclei. Subsequently, the number of viable cells from the FITC images and the number of dead cells from the TexasRed images were determined.

### Nucleic acid extraction and quantification

Total RNA was extracted from cell lines with TRIZOL solution (Invitrogen) according to the Chomczynski's procedure.[27] Obtained RNA was purified from DNA residues with the DNA-free DNA Removal Kit (Ambion) following the manufacturer's instructions. The procedure was completed by examining the quantity and quality of the resulting RNA solution. The concentration was measured spectrophotometrically at a wavelength of λ = 260 nm by NanoDrop 2000. The degree of possible material degradation was verified by an electrophoretic separation in the 1% agarose gel. Purified RNA was a template for complementary DNA (cDNA) synthesis. Reverse transcription was performed using 500 ng of material with the Transcriptor High Fidelity cDNA Synthesis Kit (Roche) according to the attached procedure.

### qRT-PCR

The quantitative reverse transcriptase real-time PCR (qRT-PCR) was performed with the LightCycler480 using LightCycler480 Probes Master and Universal Probe Library (UPL) Set for Human (Roche). Primers with probes were designed by the Universal Probe Library Assay Design Center (https://qpcr.probefinder.com/organism.jsp). Primers sequences are given in Table 1. The efficiency of primers was estimated on the standard curve with series of 2-fold dilutions. The qRT-PCR proceeded under the following conditions: an initial 5 min preincubation at 95°C, 45 cycles of denaturation in 95°C for 10 sec, annealing at 55°C for 30 sec and extension at 72°C for 10 sec. Hypoxanthine phosphoribosyltransferase (HPRT) was used as the endogenous control. The LightCycler480 Software release 1.5.1.62 allowed for an analysis basing on the E-method (Roche) expression level. All experiments were performed in triplicates.

**Table 1:** Primer sequences for qRT-PCR.

Primer Sequence 5’ → 3’	UPL
RIG1_L GGCAAGTCCCGCTGTAAAC	42
RIG1_R TTGGTATCTCCTAATCGCAAAAG	
OAS1_L CATCCGCCTAGTCAAGCACT	87
OAS1_R CAGGAGCTCCAGGGCATAC	
OAS3_L TCCCATCAAAGTGATCAAGGT	41
OAS3_R ACGAGGTCGGCATCTGAG	
TLR3_L TGGATATCTTTGCCAATTCATCT	80
TLR3_R ATCTTCCAATTGCGTGAAAAC	
INTγ_L GGCATTTTGAAGAATTGGAAAG	21
INTγ_R TTTGGATGCTCTGGTCATCTT	
GPX_L CAACCAGTTTGGGCATCAG	77
GPX_R TCTCGAAGAGCATGAAGTTGG	
TNC_L CCGGACCAAAACCATCAGT	76
TNC_R GGGATTAATGTCGGAAATGGT	
HPRT_L TGACCTTGATTTATTTTGCATACC	73
HPRT_R CGAGCAAGACGTTCAGTCCT	

### Western blot analysis

Transfected cells were lysed by sonication. 150 μg and 10 μg of denaturated protein extracts were separated on 12% and 15% SDS-PAGE (SDS-polyacrylamide gel electrophoresis) for tenascin-C and glyceraldehyde 3-phosphate dehydrogenase (GAPDH) detection respectively. Spectra Multicolor Broad Range Protein Ladder (Thermo Fisher Scientific) was used as the size marker. Subsequently the wet transfer onto PVDF membrane with 0.45 μm pore size (GE Healthcare) was performed in transfer buffer (25 mM Tris base, 190 mM glycine, 20% methanol). The membranes were placed in the SNAP i.d. 2.0 apparatus (EMD Milipore), where it was blocked with a 0.5% solution of skimmed milk in PBS. For detection of tenascin-C the TNC polyclonal antibody (H-300, Santa Cruz) was used, while GAPDH was detected using monoclonal antibody (0411, Santa Cruz). Antibodies were diluted 1:500 in 3% BSA (Sigma-Aldrich). Membranes were incubated with the primary antibody for 10 min or overnight,. Afterwards, secondary anti-rabbit IgG and anti-mouse IgG antibodies conjugated with horseradish peroxidase (HRP) (Sigma-Aldrich) were used. Detection of a protein was carried out with WesternBright Sirius Chemiluminescent Detection Kit (Advansta). Intensity of individual bands was analyzed qualitatively by Multi Gauge ver. 2.0 (Fujifilm).

### Cell proliferation and migration assays

Real-time cell proliferation and migration were monitored by measuring changes in electrical impedance using xCELLigence RTCA DP system (ACEA Biosciences). In the first step, the background impedance of a culture medium in plates was measured. In proliferation experiments, the 16-well plate with incorporated sensor electrode arrays (E-plate) were seeded with 10,000 cells per well in a final volume of 200 **μ**L of medium. Next, the impedance was measured at 15-minute intervals for 72 h. The transfection was performed 24 h after cell seeding. For migration assays, the 16-well plate consisting of two chambers with a microporous polyethylene terephthalate (PET) membrane containing microfabricated gold electrode arrays on the bottom side of the membrane between them (CIM-plate) was used. 10,000 cells per well were seeded in an upper chamber in unsupplemented medium and moved towards a bottom chamber filled with supplemented medium. The impedance was measured at 15-minute intervals for 96 h. The seeded cells were transfected 24 h before experiment. Obtained cell index (CI) values were entered to the GraphPad Prism ver. 5.1 (GraphPad Software, Inc., La Jolla, CA, USA) software and used to calculate half maximal inhibitory concentrations (IC_50_) of effecting cell proliferation. In case of transwell migration assay, obtained CI values from each experimental condition were plotted against time, fitted to four-parameter logistic non-linear regression model and the half-time of migration (half maximal effective time, ET_50_) was calculated.

### Scratch assay

Cell migration scratch assay was performed on 12-well plate, with 200,000 cells per well seeded in a supplemented medium. Cells were grown for 24 h under optimal growth conditions. Next, the transfection was performed and the plate was incubated for another 24 h. After this time the medium was replaced and "wound" was created in the monolayer of cells covering the well. The scar effect was obtained by scraping cells in a straight line using a 200 μL tip. From that moment, images of the cultured cells at 12 h intervals were taken for 48 h using a fluorescence microscope at 5x magnification. The analysis of the degree of scarring of individual "wounds" was carried out by computer using the Tscratch program (CSElab).

### Statistical analysis

Experimental results were subjected to statistical evaluation using GraphPad Prism ver. 5.1. Values presented are an average of three biological along with three technical replicates as mean values ± standard error of the mean (SEM). Differences between mean values of tested samples and controls were compared with the analysis of variance (ANOVA) extended by Tukey or Bonferroni post-hoc tests. Statistically significant results were obtained at the level of: * for p<0.05; ** for p<0.01; *** for p<0.001; no statistical significance for p≥ 0.05.

## Results and discussion

### Synthesis and characterization of nanoparticles

**Fig 1:**
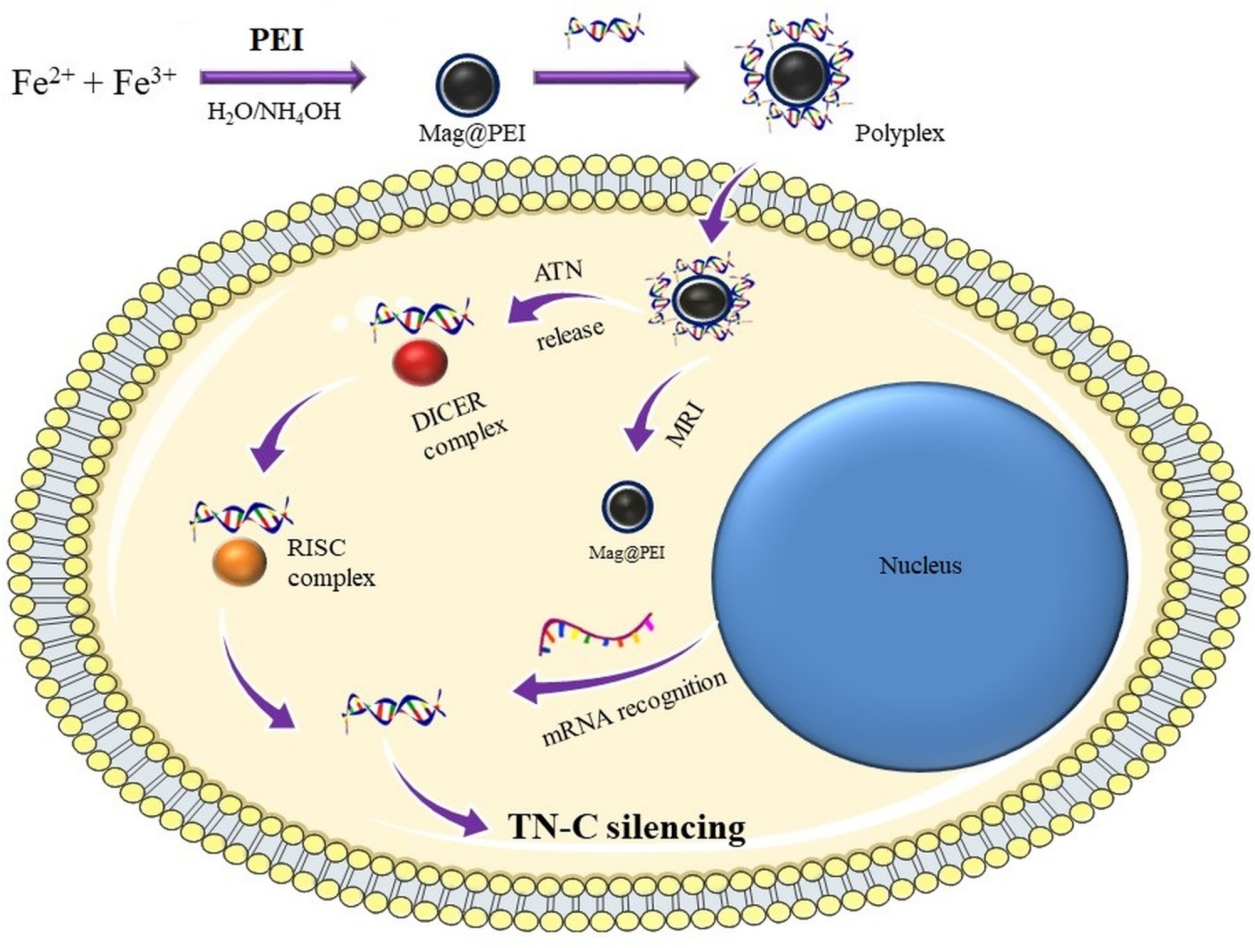
Schematic diagram of preparation of Mag@PEI /ATN-RNA complexes and its application in RNAi therapy of GBM cells.

In the first step (Fig 1), the PEI coated magnetic nanoparticles (Mag@PEI) were prepared in a straightforward manner *via* previously reported procedure in one step protocol from iron III and II chlorides in the molar ratio 2:1 in the presence of polyethyleneimine and surfactant.[25]

**Fig 2:**
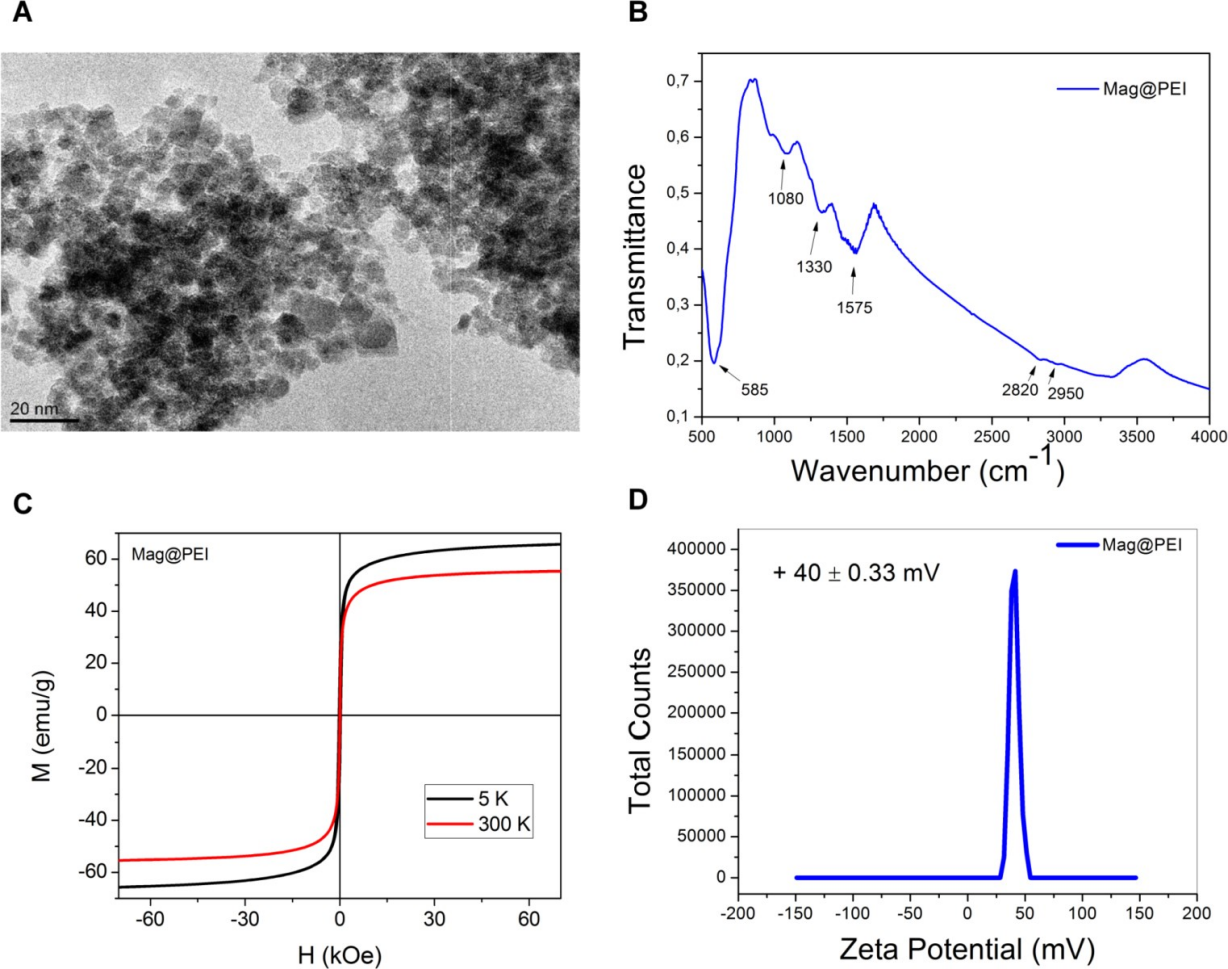
Characteristics of Mag@PEI. A. TEM picture of Mag@PEI NPs B. FTIR spectra of Mag@PEI NPs C. Hysteresis loop recorded for Mag@PEI NPs by means of SQUID D. Zeta potential of synthesized Mag@PEI NPs.

The morphology of obtained nanocomposites was investigated by means of TEM. The particles were spherical in shape, however, some aggregations were observed (Fig 2A). The obtained average size of magnetic nanomaterials ranged between 8 to 12 nm. In order to confirm the successful coating with PEI, the FT-IR spectrum was recorded. The spectrum showed a peak at 585 cm^**-1**^ which was assigned Fe-O bond. Characteristic signals at 1080 cm^**-1**^ and 1330cm^**-1**^ were attributed to C-N stretching vibration from the PEI. The peak observed at 1575cm^**-1**^ corresponds to N-H bending vibration from amine moieties present in polyethylenimine structure. Moreover, the signals from CH_**2**_ groups were observed at 2820 cm^**-1**^ and 2950 cm^**-1**^, respectively. Thus, FT-IR proved attachment of PEI at the surface of magnetic nanoparticles (Fig 2B).

Since PEI molecules bound to magnetite nanoparticles by electrostatic interaction and the negative charge on the surface of the particles is converted to positive charge, zeta potential of Mag@PEI NPs was measured. Performed studies revealed that zeta potential of Mag@PEI NPs was +40.6 mV what indicated the high colloidal stability of nanocomposites and additionally confirmed successful functionalization of magnetic nanoparticles (Fig 2D). Encouraged by the results obtained from zeta potential measurement we also investigated the colloidal stability of Mag@PEI NPs in real-time by Multiple Light Scattering (MLS) using Turbiscan apparatus.

This technique provides the information about TSI global index which is a sum of all destabilization processes occurring in the sample. However, one needs to take into account that the lower the TSI index, the more stable the sample. The investigation of kinetic destabilization for Mag@PEI NPs in water was monitored over a period of 24 h. The TSI index changed over time from 0.6 to 6.8 for 4 h and 24 h, respectively, what proved that the Mag@PEI NPs had high colloidal stability and sedimentation process occurred only in small extent (Figure A in S1 File). The magnetic properties of obtained materials were measured by means of SQUID. The sample exhibited superparamagnetic behavior, as evidenced by the lack of hysteresis loop and blocking temperature of 155 K. Obtained nanomaterial had high magnetic saturation above 40 emu/g at 300 K, which is important for its further guidance by external magnetic field and during magnetic separation. Moreover, the sample was easy handled by an external magnet (Fig 2C).

**Fig 3:**
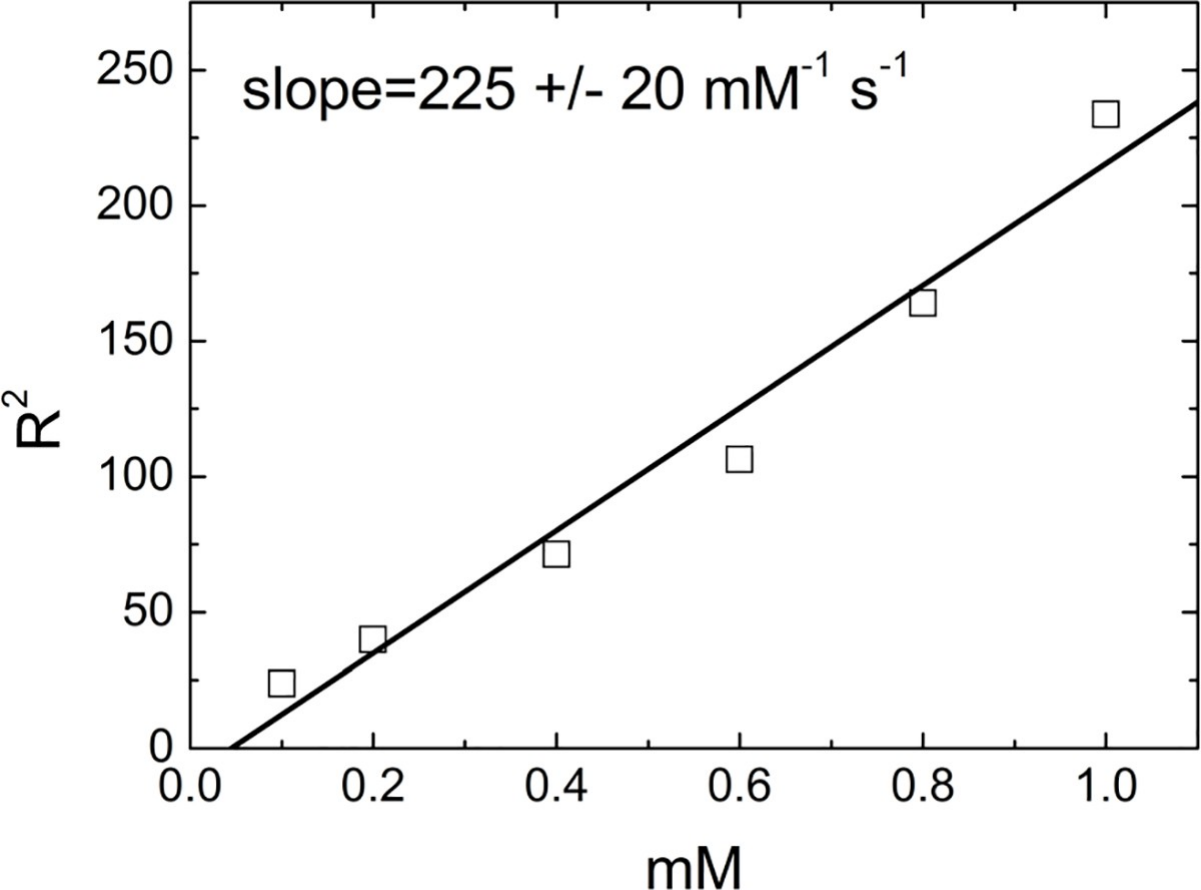
Contrast properties of Mag@PEI. Relaxation rates R^**2**^ as well as relaxivity obtained from MRI experiment for Mag@PEI NPs in agarose gel 2 wt %.

Furthermore, contrast properties of synthesized magnetic nanocarriers and their potential application in magnetic resonance imaging were assessed. In order to avoid drift of magnetic nanomaterial in the high magnetic field, the suspensions of nanoparticles in an agarose gel (2 mg/mL) were prepared, according to recently reported protocol.[28] The spin echo (MEMS) imaging results obtained for Mag@PEI NPs are shown in Fig 3. The relaxivity value measured for our nanocarrier was as high as 225 mM^**-1**^s^**-1**^ proving high contrast properties of synthesized nanoparticles. Moreover, this value is much higher than the relaxivity values reported for commercial contrast agents based on magnetic nanoparticles like Feridex (120 mM^**-1**^s^**-1**^), Resovist (186 mM^**-1**^s^**-1**^) and Combidex (65 mM^**-1**^s^**-1**^).[29] Thus, our nanoparticles exhibited strong application potential in further MRI studies.

**Fig 4:**
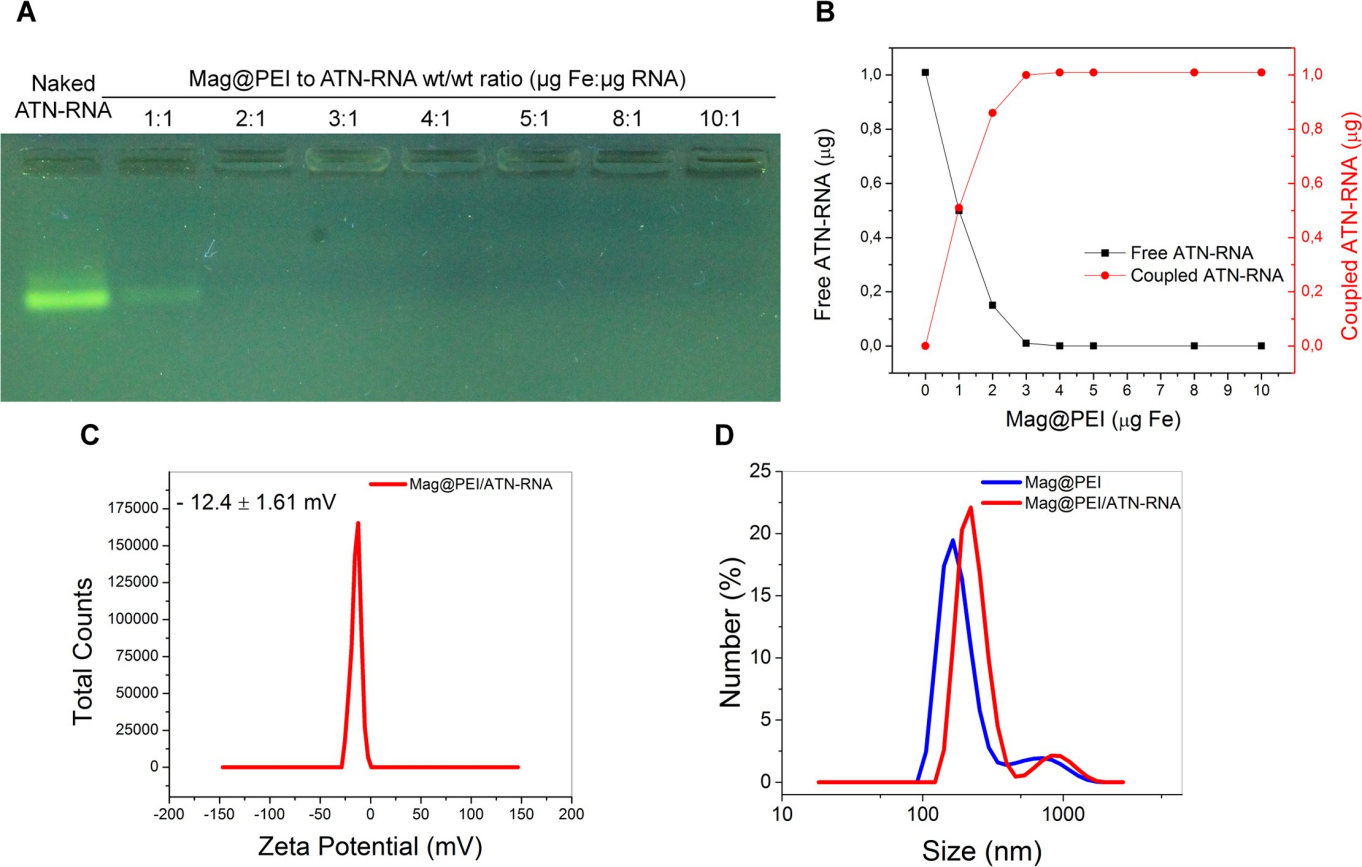
Binding of ATN-RNA to Mag@PEI NPs. A. Agarose gel electrophoresis of Mag@PEI/ATN-RNA complexes at the different mass ratio. B. Binding capability of Mag@PEI NPs towards ATN-RNA recorded using Nanodrop. C. Zeta potential of complexes obtained between Mag@PEI NPs and ATN-RNA at ratio 3:1. D. DLS size distribution for Mag@PEI NPs and Mag@PEI/ATN-RNA complexes at ratio 3:1.

The characterized nanocarrier was further used to determine its binding capability towards ATN-RNA. In this experiment, ATN RNA was mixed with Mag@PEI NPs for 30 minutes at different weight ratios varied from 1:1 to 10:1. We expected that negatively charged ATN-RNA will interact with positively charged Mag@PEI NPs resulting in polyplexes formation. This type of structure is often postulated in literature when PEI is used as a carrier for nucleic acids delivery.[30] In the next step, obtained Mag@PEI/ATN-RNA complexes were submitted to agarose gel electrophoresis assay to visualize the linking of ATN-RNA to Mag@PEI NPs (Fig 4A). It is worth to highlight that the total amount of iron in the sample was first determined to keep this value constant in order to repeat the experiments with different batches of nanoparticles which could slightly differ between each other. Analysis revealed that 2 weight equivalents were sufficient to bind almost all of the ATN-RNA used in the experiment. However, to get a deeper insight into this process, ATN-RNA concentration in the supernatant was investigated by UV-Vis spectroscopy. This technique has higher sensitivity than the gel electrophoresis. The data are presented in Fig 4B. UV-Vis experiments revealed that at Mag@PEI NPs to ATN-RNA ratio of 2:1, free ATN-RNA was still present in the supernatant and the loading was around 90%. However, at the ratio 3:1, the ATN-RNA was completely linked to Mag@PEI NPs. Furthermore, at this ratio, a change in zeta potential from +40 mV for Mag@PEI NPs to –12 mV for Mag@PEI/ATN-RNA complexes was observed (Fig 4C). This clearly proved that negatively charged ATN-RNA bind to the surfaces of Mag@PEI NPs.

The DLS measurements were performed to investigate the size of synthesized nanoparticles and prepared complexes in water. The Mag@PEI NPs had the hydrodynamic diameter of ~150 nm while the hydrodynamic diameter of Mag@PEI/ATN-RNA complexes increased slightly to around 200 nm after nucleic acid binding (Fig 4D).

**Fig 5:**
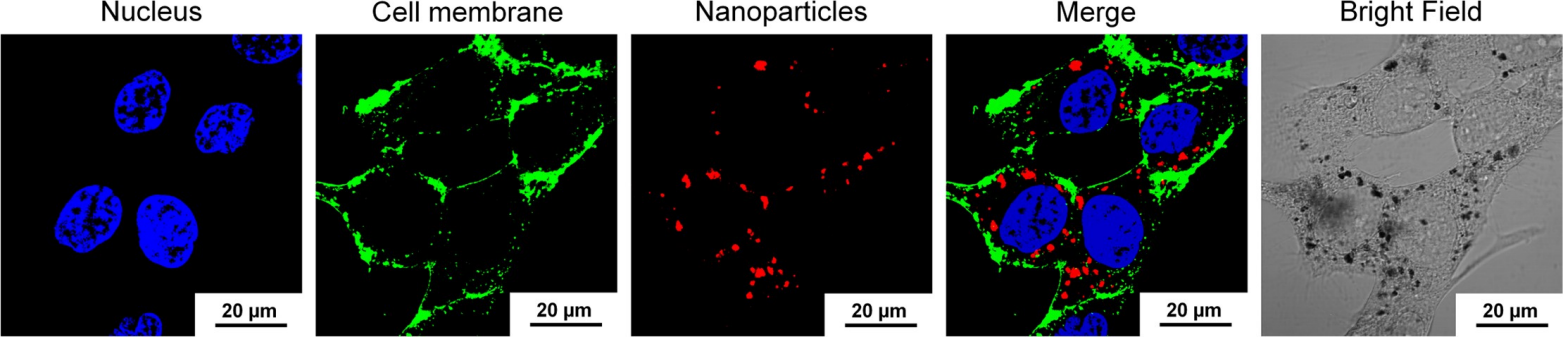
Cellular uptake of complexes containing fluorescently labelled Mag@PEI NPs into U-118 cells. The representatives of the colors are blue (Hoechst 33342) for nuclei, green (Concanavalin A-FITC) for cell membranes, and red (ATTO550) for Mag@PEI nanoparticles.

The uptake of Mag@PEI/ATN-RNA complexes by U-118 cells was investigated by means of confocal microscopy. First, Mag@PEI nanoparticles were labelled with ATTO 550 dye. As illustrated in Fig 5, glioma cells could be successfully transfected with nanoparticles since the red fluorescence from the dye was observed in the cytoplasm. Moreover, the analysis of the 3D reconstruction further confirmed their internalization capacity of obtained complexes. (S1 Video). The bright field images show the presence of black dots corresponding to the aggregated complexes containing fluorescently labelled Mag@PEI NPs.

**Fig 6:**
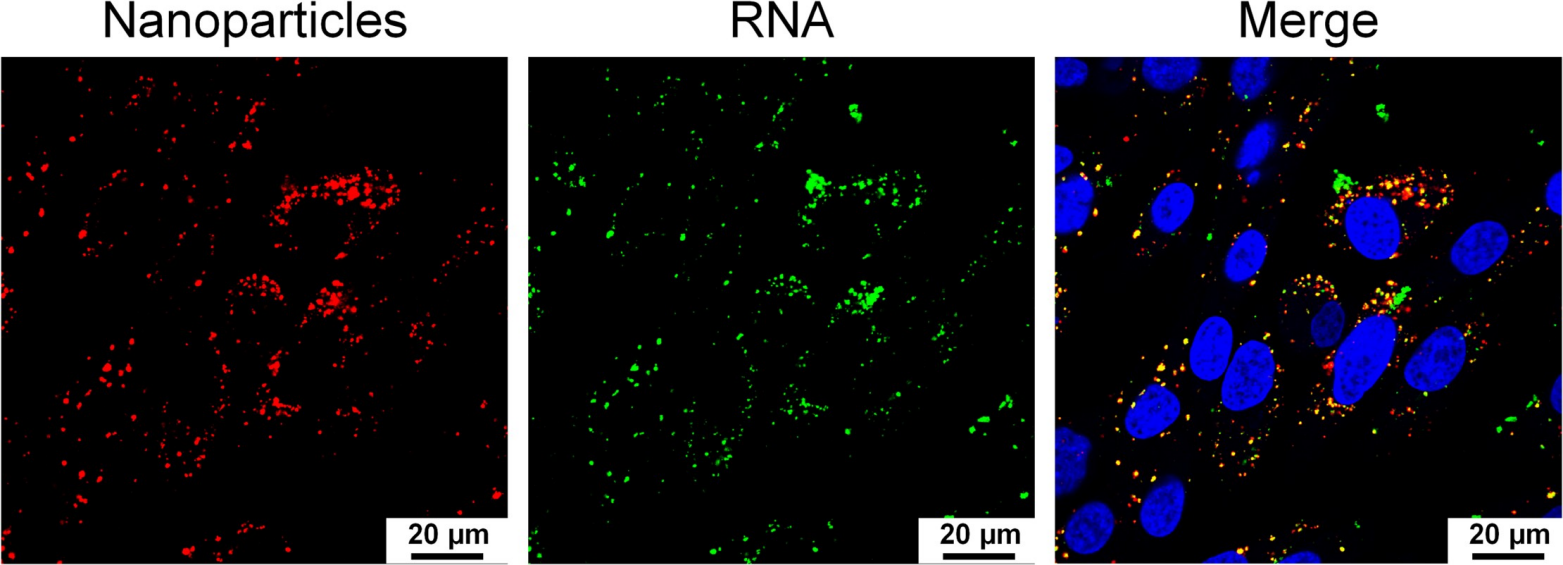
Colocalization of Mag@PEI NPs and ATN-RNA in U-118 cells. The representatives of the colours are blue (Hoechst 33342) for nuclei, green (FITC) for ATN-RNA, and red (ATTO550) for Mag@PEI nanoparticles.

A colocalization analysis was also performed to further demonstrate transfection of Mag@PEI/ATN-RNA complexes into glioblastoma cells. As shown in Fig 6, there is a visible colocalization between the ATTO 550 labelled Mag@PEI nanoparticles (red color) and the FITC labelled ATN-RNA (green color) in the cytoplasm suggesting high transfection efficiency of the synthesized nanoparticles.

### Cytotoxicity of magnetic nanocarierr and complexes with ATN-RNA

**Fig 7:**
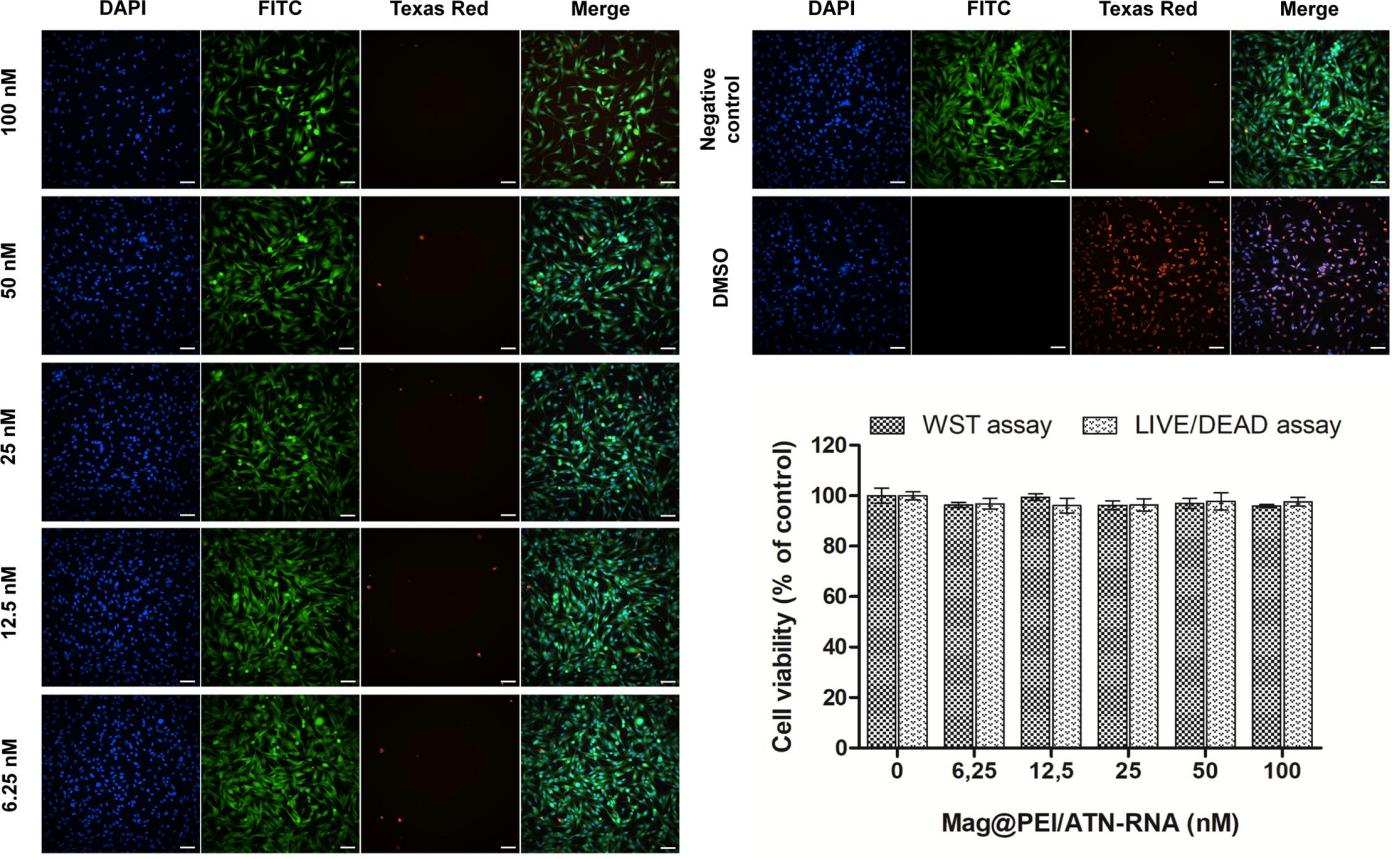
Representative high-content images of U-118 cells exposed to Mag@PEI/ATN-RNA complexes (6.25–100 nM). 10% DMSO was used as a positive control. Images were obtained using different filters to detect nuclei (DAPI), live cells (FITC), and dead cells (TexasRed). The scale bars denote 100 μm. Cell viability of U118 cells exposed to Mag@PEI/ATN-RNA complexes (6.25–100 nM) for 24 h. The value at X-axis in Fig 7 corresponds to concertation of free ATN-RNA on the carrier.

Cytotoxicity of both Mag@PEI NPs and polyplexes bearing ATN-RNA on GBM U-118 cells were determined by two cytotoxicity tests, namely WST-1 and LIVE/DEAD assays. In the first step, the toxicity of Mag@PEI NPs without bounded ATN-RNA at the concentrations of NPs corresponding to the concentrations used in further transfection experiments (Figure B in S1 File). Mag@PEI carrier did not cause any significant toxic effect in the investigated concentration range and the data from both tests were consistent. Also, SRB test did not show any toxicity of our carrier (Figure C in S1 File). Subsequently, we assessed the cytotoxicity of Mag@PEI/ATN-RNA complexes (Fig 7). To keep the same range of concentrations as previously used in RNAi approach [10], we used the ATN-RNA concentration between 10 to 100 nM. Both cytotoxicity assays revealed high viability of glioblastoma cells in the investigated concertation range. So we could exclude undesired side effects coming from the nanoparticles.

### Immune response

**Fig 8:**
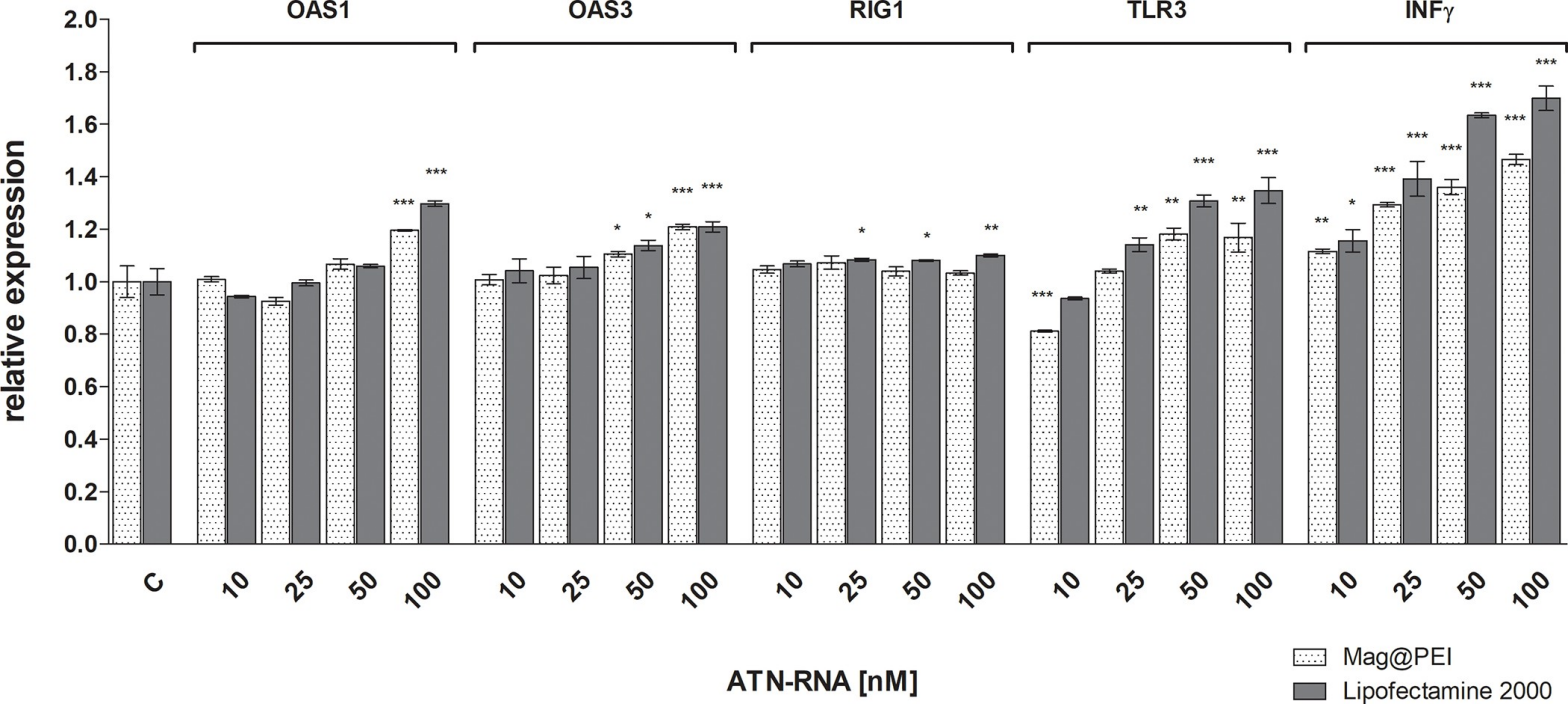
The expression level of immune response genes after lipo- and nano-mediated ATN-RNA delivery to U-118 cell line. The relative expression level of the expression of OAS1, OAS3, RIG1, INFγ and TLR3 established by qRT-PCR calculated wit the –ΔΔCp method. Statistical evaluation of ATN-RNA versus control (Clipo or Cnano, respectively) cells was performed using one-way ANOVA followed by Tukey’s posthoc test. Significance value: * p< 0.05, ** p< 0.01, *** p< 0.001 compared to untreated cells (Clipo or Cnano, respectively). Legend: Mag@PEI- nano-mediated ATN-RNA delivery; Lipofectamine—lipo mediated ATN-RNA delivery.

Taking into account the possible use of nanoparticles as the therapeutic tool in GBM treatment, the analysis of expression level of the genes involved in innate immune response was performed. We examined whether our nanoparticles delivery system would elicit an innate immune response *in vitro*. Undesired immune responses are an important consideration in gene therapy and in the development of gene delivery systems because the introduction of exogenous genes can activate the innate immune system of human cells to combat foreign gene or pathogen invasion.[31] For example, siRNA can provoke an immune response *via* its interactions with Toll-like receptors (TLRs) and trigger an interferon (IFN) response.[32] Additionally, systemically introduced lipid nanoparticles have been reported to induce an immune response in mice.[33] Thus, the potential immunostimulatory properties of a proposed gene delivery system are important. We characterized U-118 GBM cells for their innate immune response to the nanoparticle complexes. We measured the expression of OAS1, OAS3, RIG1, TLR3 and INFγ using the qRT-PCR (Fig 8). The changes after ATN-RNA treatment were either not observed, as for the RIG1 gene or negligible, as for OAS3 gene. However, the highest concentration of ATN-RNA (50, 100 nM) caused the weak activation of interferon response of above mentioned genes, but this activation was slightly higher in case of lipofection when compared to magnetic nanocarrier—about 8% (p<0.01 and p<0.005, respectively). The higher activation of OAS 1 gene was measured both in case of lipofectamine and nanoparticles treatment, but it needs to be highlighted, that at 100nM ATN-RNA working concentration the expression level of that gene was about 30% higher for lipofection, what gives 10% increase in relation to the nanoparticles-mediated delivery (p<0.005).

The highest activation with ATN-RNA both with lipofectamine and using Mag@PEI nanocarrier was observed for TLR3 and INFγ genes. The ATN-RNA delivered with lipofectamine caused the TLR3 gene expression activation at the level from 16% to 37%, starting from the 25 nM ATN-RNA concentration (p<0.005). The Mag@PEI NPs used as the ATN-RNA carrier resulted in the lowest activation of the TLR3 gene expression with the observed 8–15% level, for 25–100 nM ATN-RNA concentrations (p<0.01).

INFγ gene was significantly upregulated both by the lipofectamine and Mag@PEI NPs. However, with the same ATN-RNA concentration, the gene expression was elevated at the higher level by the lipofectamine-mediated ATN-RNA delivery. With the already lowest concentration, we observed 10–12% overexpression by the lipofection and Mag@PEI NPs (p<0.01). However, starting from the 25 nM ATN-RNA concentrations we noticed the growing differences with the effect of lipofectamine versus Mag@PEI NPs. With this concentration, we observed the INFγ gene overexpression of about 27 and 42% for Mag@PEI NPs and lipofection, respectively. The highest concentrations caused –for 50 nM: 28 and 63%, for 100 nM: 44 and 75% in case of use of Mag@PEI NPs and lipofection, respectively.

### RNA interference therapy

**Fig 9:**
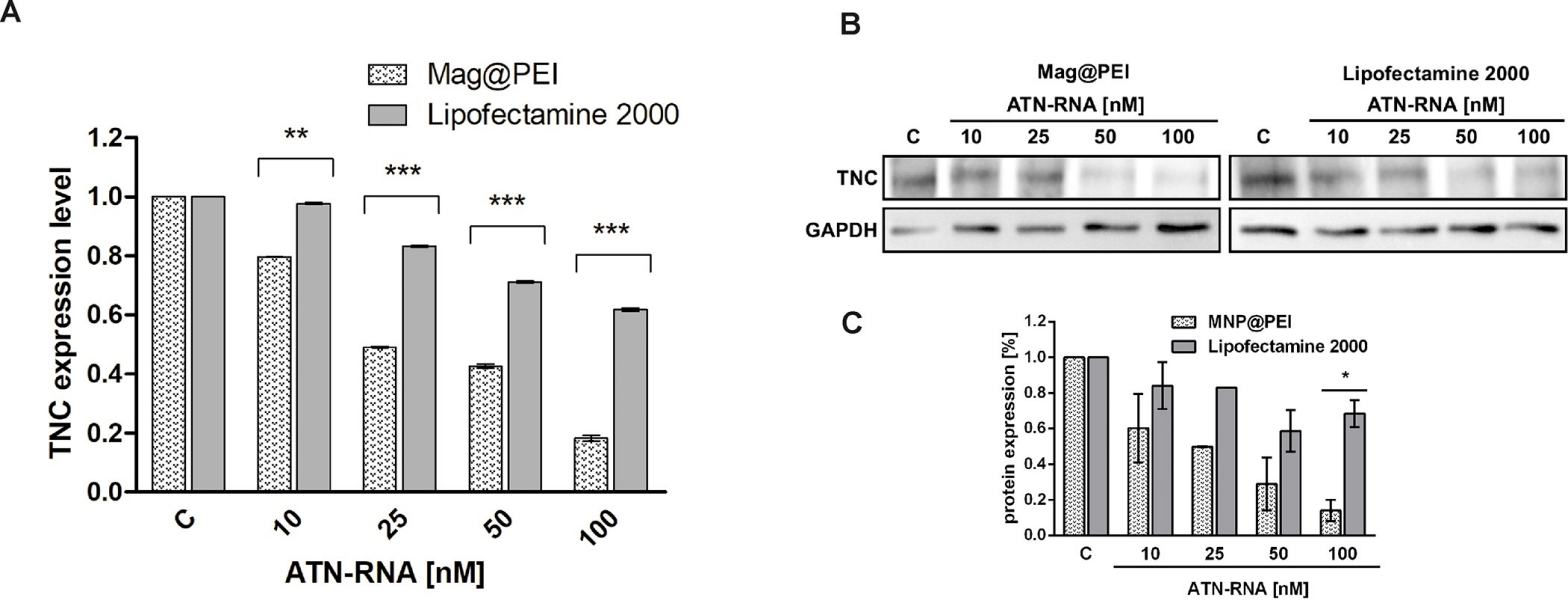
The expression level of TN-C after lipo- and nano-mediated ATN-RNA delivery to U-118 cell line. A. The relative expression level of the expression of TN-C established by qRT-PCR calculated with the ΔΔCp method. B. The protein expression level measured by Western blot with densitometric analysis (C.) Statistical evaluation of ATN-RNA versus control (Clipo or Cnano, respectively) cells was performed using one-way ANOVA followed by Tukey’s posthoc test. Significance value: ** p< 0.01, *** p< 0.001. Legend: Mag@PEI- nano-mediated ATN-RNA delivery; Lipofectamine- lipo mediated ATN-RNA delivery.

To achieve down-regulation of TN-C expression in U-118 glioblastoma cell line, lipofection along with Mag@PEI NPs with various concentration of ATN-RNA– 10, 25, 50 and 100 nM was performed. 24 hours after transfection the expression level of tenascin-C was examined by qRT-PCR analyses. The downregulation of TN-C mRNA expression was observed compared to controls treated with the transfection agent or with scrambled RNA. The level of TN-C was decreased from 2% at a concentration of 10 nM ATN-RNA to 34% for cells treated with 100 nM ATN-RNA in comparison to the untreated cells (p< 0.005) in case of samples where ATN-RNA was delivered with lipofectamine (Fig 9). At the same time, the above mentioned range of ATN-RNA concentration was delivered with Mag@PEI nanoparticles resulted in the significant drop of the TN-C expression level. The downregulation effect was visible already with the lowest concentration with the decrease at 20%. The highest concentrations: 25, 50 and 100 nM caused the TN-C downregulation about 43, 48 and 80%, respectively (p< 0.005) (Fig 9A).

The qRT-PCR analysis was also supported by the direct analysis of the protein expression level. The Western blot detection reveled again with the more significant drop of the TN-C expression caused by the nanoparticles-mediated ATN-RNA delivery. We observed already 40% decrease of the protein expression with the 10 nM ATN-RNA. The highest concentration used caused the dramatic drop of the protein expression measured as the 85% of the decrease. The lipofectamine mediated ATN-RNA delivery at the same time caused only about 40% decrease of the expression level with ATN-RNA concentrations 50 and 100 nM (Fig 9B). These observations were fully consistent with the obtained relative TN-C expression level measured by qRT-PCR.

These results clearly support the idea of nanoparticles mediated delivery in order to achieve the significant biological effect along with the lowest concentration of therapeutic agent. The Mag@PEI mediated ATN-RNA delivery was already efficient with the lowest- 10 nM concentration- 20% of TNC downregulation, while the lipofectamine at the same time was not able yet to deliver the RNA. During the whole experiment, we observed about 30–50% higher efficiency of nanomediated delivery compared to the lipofectamine-driven one.

### Proliferation and migration of GBM cells

**Fig 10:**
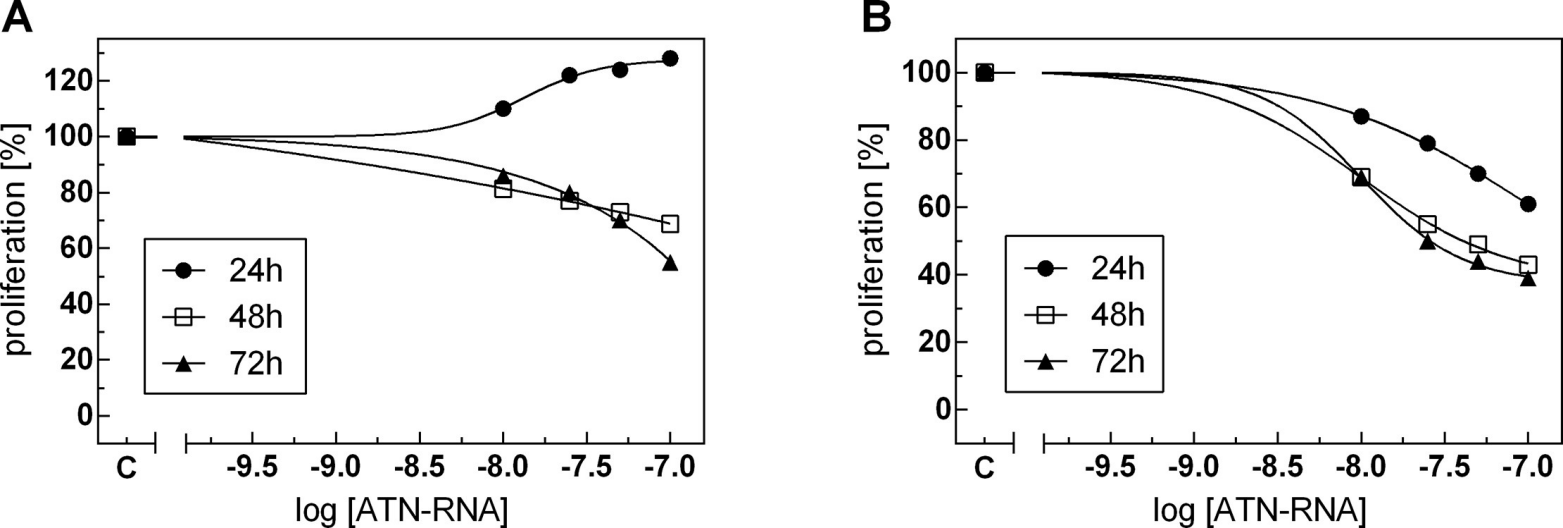
**Anti-proliferative activity of ATN-RNA, after nano- (A) and lipo-mediated (B) delivery.** Proliferation was monitored in real-time using the xCELLigence system. Differences between CI values for ATN-RNA treated and control cells were statistically evaluated using one-way ANOVA followed by Tukey’s posthoc test. Dose-dependent effects of ATN-RNA on proliferation was evaluated using non-linear regression by fitting experimental values to sigmoidal, bell-shaped equation. Legend: Mag@PEI- nano-mediated ATN-RNA delivery; Lipofectamine—lipo mediated ATN-RNA delivery.

In order to investigate the delivery efficacy of the carriers, we measured the cells proliferation rate. U-118 glioblastoma cell line was treated with Mag@PEI NPs and Lipofectamine combined with ATN-RNA followed by the real-time cell proliferation assay with the xCELLigence system. The cell's ability for proliferation was measured for 72 h. We noticed ATN-RNA concentration-dependent decrease of proliferation rate both for Lipofectamine carrier with the significant decrease of proliferation with 100 nM–ATN-RNA concentration. The proliferation rate dropped for ATN-RNA delivered with the cationic carrier from 40–61% (Fig 10B). However, for Mag@PEI NPs we observed the opposite effect, resulting in the increase of proliferation- most effective concentration of ATN-RNA was 100 nM, with an increase from 10–28% after 24 h, respectively (Fig 10A). Noteworthy, 25 nM and 50 nM of ATN-RNA was already sufficient concentration for the efficient changes of GBM cells proliferation. After 48 and 72 h post transfection we observed again the inhibition of proliferation, from 7–45%, respectively. This allowed us to assume that the most effective delivery of ATN-RNA is achieved after first 24 h from transfection. We also would postulate that the significant drop of the proliferation rate both for Lipofectamine and for nanoparticles mediated delivery 48 and especially 72 h post transfection is more likely due to the simple death of the cells, rather than the ATN-RNA action.

To get more insight into the down-regulation of TN-C expression by ATN-RNA on the mobility of glioblastoma cancer cells, real-time measurements of migration was carried out. In this approach, U-118 cells were treated again with Mag@PEI NPs and Lipofectamine and ATN-RNA and allowed to migrate through microporous polyethylene terephthalate (PET) membrane towards chemoattractant. The number of cells that crossed the membrane was continuously recorded.

**Fig 11:**
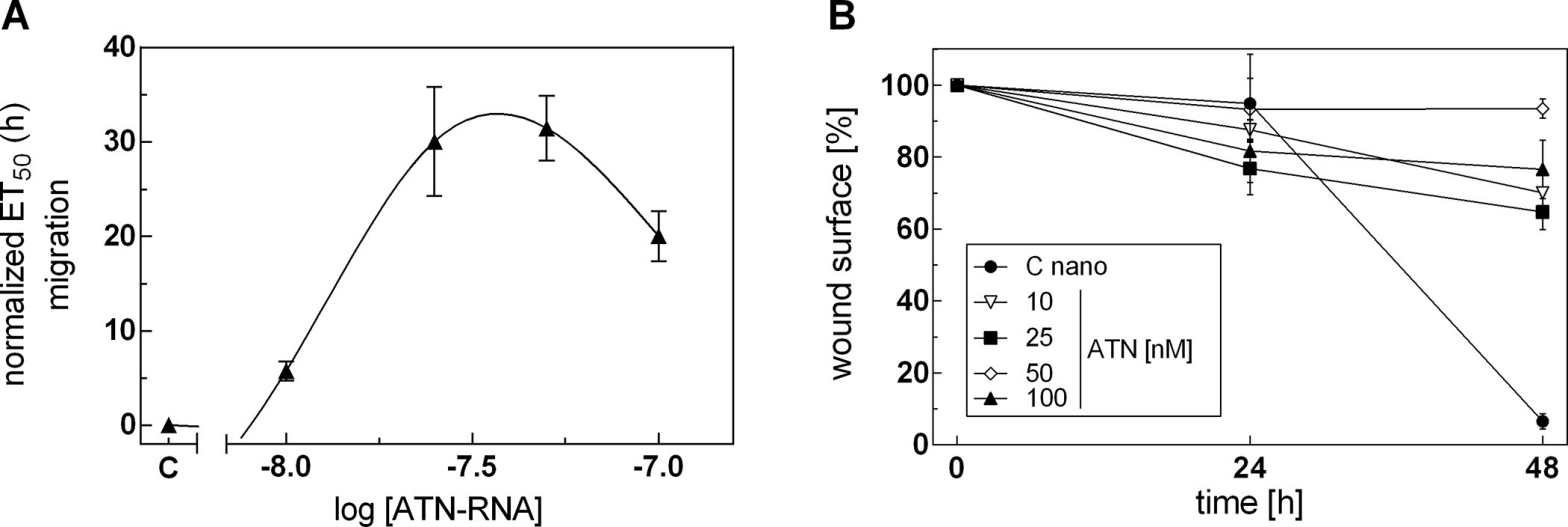
The effect of nano-mediated delivery of ATN-RNA on the migration processes. (A) Migration of U-118 GBM cancer cells was studied using the xCELLigence system. Serum-depleted cells were transfected with increasing concentrations of ATN-RNA (from 10 to 100 nM) or vehicle (Opti-MEM, C-control). Impedance (CI values) of each experimental condition was recorded over time, plotted against time, fitted to four-parameter logistic non-linear regression model and ET50 was calculated for each ATN-RNA concentration to generate dose-response curves. The ET50 value was normalized to the data obtained for cells treated with native Mag@PEI and plotted as normalized half maximal effective time (ET50) of cell migration against ATN-RNA concentrations. (B) The scratch assay analysis. U-118 GBM cells were transfected with Mag@PEI/ATN-RNA complexes. Images were captured after 24 and 48 h (Figure D in S1 File). The rate of migration was measured by quantifying the total distance that the U-118 cells moved from the edge of the scratch toward the centre of the scratch.

We found that down-regulation of TN-C expression by ATN-RNA delivered by Mag@PEI NPs significantly impaired the cell migration in GBM cells (Fig 11). The results were quantitatively assessed during 72 h of experiment and showed that U-118 cells transfected with ATN-RNA had the lowest motility beginning from 12 h post transfection. It was established that Mag@PEI NPs with ATN-RNA delayed the migration of U-118 cells by 5.76 ± 1.02, 30.06 ± 5.78, 31.46 ± 3.41 and 20.03 ± 2.65 h with 10, 25, 50 and 100 nM concentration, respectively (Fig 11A). Notably, the most effective concentration influencing the migration potential of the cells is 50 nM, while 100 nM seems to cause the elevated cells death. One can notice the entirely different results observed for the ATN-RNA delivery driven by Lipofectamine. During the experiment, we were not able to detect the specific migration impairment, most probably due to the high toxicity of Lipofectamine. To get more insight into the problem, we performed also the scratch assay. Again, with the lipofectamine delivered ATN-RNA we were not able to quantify the existing wound since the transfection resulted in the cells death. Conversely, the results from nanoparticles-mediated ATN-RNA delivery in the wound-healing assay demonstrated that U-118 ATN-RNA treated cells migrate into the scratched area more slowly than the control cells- 18% after 24 and 24% after 48 h, respectively (Fig 11B).

Notably, the infection did not change substantially also the viability of the cells, what was observed in terms of lipofection (Figure D in S1 File) Thus, taking together the independent experiments proved the significant impairment of migration rate of the Mag@PEI/ATN-RNA treated U-118 cells, suggesting the therapeutic potential of the used nanocomposite. This extremely interesting observation could suggest either the gradual release of the dsRNA from the nanoparticles surface, resulting in the best effect already at the higher concentration or the highest stability of dsRNAs. At the same time, one can notice the significant migration impairment is already established with the 25 nM concentration. This could suggest that the nanoparticles-mediated delivery would use the lowest concentration of the therapeutic agent allowed for an efficient silencing of the tenascin-C. Observed migration delay as the results with the nanoparticles delivery is fully consistent with the tenascin-C expression level measured by qRT-PCR. The highest TN-C downregulation has functional consequences with the migration rate of U-118 glioblastoma cells.

Our functional studies with using both nano- and lipo- carriers showed the potential of nanoparticles with dsRNA delivery. ATN-RNA in our previous studies had a great impact on the migration and proliferation properties of cancer’s cells resulted in the decrease of both of these potentials. In the present study, however, we observed surprisingly the increase of the proliferation potential caused by the ATN-RNA delivery with nanoparticles. Since we observed the enhanced TN-C downregulation followed by the ATN-RNA delivery with nanoparticles, our results seem to be in concordance with the results in intracranial xenografts.[34]

The TN-C knockdown in the tumor microenvironment modulated the behavior of a tumor stromal cells, inhibited tumor invasion, and increased tumor cell proliferation. The combination of this phenotypic phenomenon is similar to the go-or-grow glioma growth phenotype found in human neoplasms, which represent characteristic, highly proliferative tumor cores and diffuse borders with a low proliferation rate.[34]

## Conclusions

We have successfully demonstrated a new dsRNA delivery system that harnesses well- characterized magnetic nanoparticles coated with PEI to effectively silence expression of tenascin-C in glioblastoma multiforme cell line. Both mere nanoparticles and their complexes with dsRNA do not show toxicity and do not provoke undesired immune responses in U-118 GBM cell line. Additionally, we proved by confocal microscopy imaging, that they could be successfully internalized into glioblastoma cells. The most important thing is that synthesized nanocarrier was a more efficient tool in delivery ATN-RNA than routinely used Lipofectamine In consequence, the gene therapy employing ATN was improved resulting in stronger silencing of TN-C, followed by the further diminishing of migration of glioblastoma cells. Finally, since the magnetic core provided high contrast properties in MRI, therefore, synthesized nanocarrier system can be considered as the robust theranostic nanoplatforms for simultaneous gene therapy and imaging. The present approach is then the first demonstration of an effective and safe dsRNA delivery based on multifunctional nanoplatforms. The used technology is an evidence of a promising nanoparticles-based shuttle with a high potential for further clinical use in GBM treatment.

## Supporting information

S1 File (Figure A) Destabilization kinetic of Mag@PEI recorder by Turbiscan apparatus over 24 h, (Figure B) Representative high-content images of U-118 cells exposed to Mag@PEI nanoparticles at various concentrations. 10% DMSO was used as a positive control. Images were obtained using different filters to detect nuclei (DAPI), live cells (FITC), and dead cells (TexasRed). The scale bars denote 100 **μ**m. Cell viability of U118 cells exposed to Mag@PEI, (Figure C) Cytotoxicity SRB of Mag@PEI for various nanoparticles concertation, (Figure D) Schratch test.
(DOCX)

S1 Video MNP@PEI_RNA_5well_3_3d. (AVI)
