# Supplementary material for: Nano-mediated delivery of double-stranded RNA for gene therapy of glioblastoma multiforme
Source: PLoS One. 2019 Mar 19;14(3):e0213852. doi: 10.1371/journal.pone.0213852 (PMC6424419; doi:10.1371/journal.pone.0213852)
Supplement: S1 File — (Figure A) Destabilization kinetic of Mag@PEI recorder by Turbiscan apparatus over 24 h, (Figure B) Representative high-content images of U-118 cells exposed to Mag@PEI nanoparticles at various concentrations. 10% DMSO was used as a positive control. Images were obtained using different filters to detect nuclei (DAPI), live cells (FITC), and dead cells (TexasRed). The scale bars denote 100 μm. Cell viability of U118 cells exposed to Mag@PEI, (Figure C) Cytotoxicity SRB of Mag@PEI for various nanoparticles concertation, (Figure D) Schratch test. (DOCX) [file pone.0213852.s001.docx]

**Nano-Mediated Delivery of Double-Stranded RNA for Gene Therapy of Glioblastoma Multiforme**

Małgorzata Grabowska^1^, Bartosz F. Grześkowiak^2^, Kosma Szutkowski^2^, Dariusz Wawrzyniak^1^, Paweł Głodowicz^1^, Jan Barciszewski^3^, Stefan Jurga^2^, Katarzyna Rolle^1,4^*, Radosław Mrówczyński^2^*

^1^ Department of Molecular Neurooncology, Institute of Bioorganic Chemistry Polish Academy of Science, Poznan, Poland

^2^ NanoBioMedical Centre, Adam Mickiewicz University in Poznan, Poznan, Poland

^3^ Department of Epigenetics, Institute of Bioorganic Chemistry Polish Academy of Science,

Poznan, Poland

^4^ Centre for Advanced Technologies, Poznan, Poland

Correspondence to: [radoslaw.mrowczynski@amu.edu.pl](mailto:radoslaw.mrowczynski@amu.edu.pl), kbug@man.poznan.pl

**
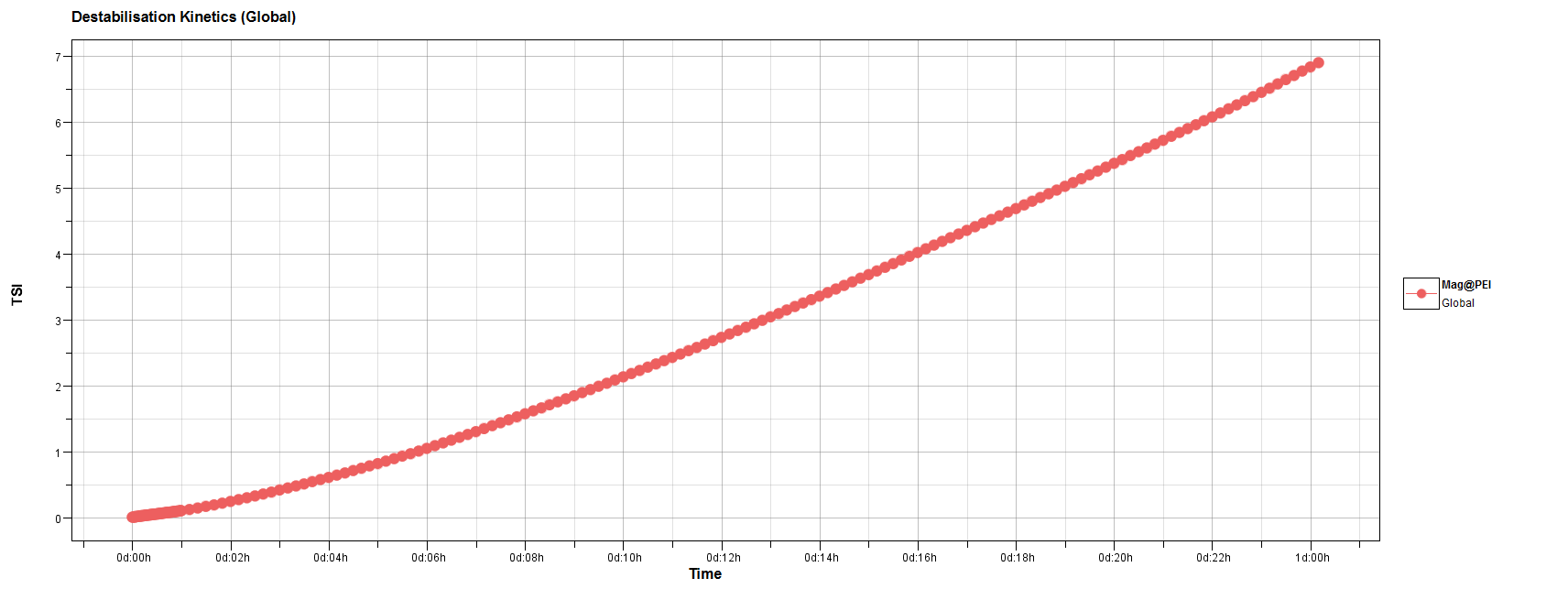
**

Figure A Destabilization kinetic of Mag@PEI recorder by Turbiscan apparatus over 24 h.


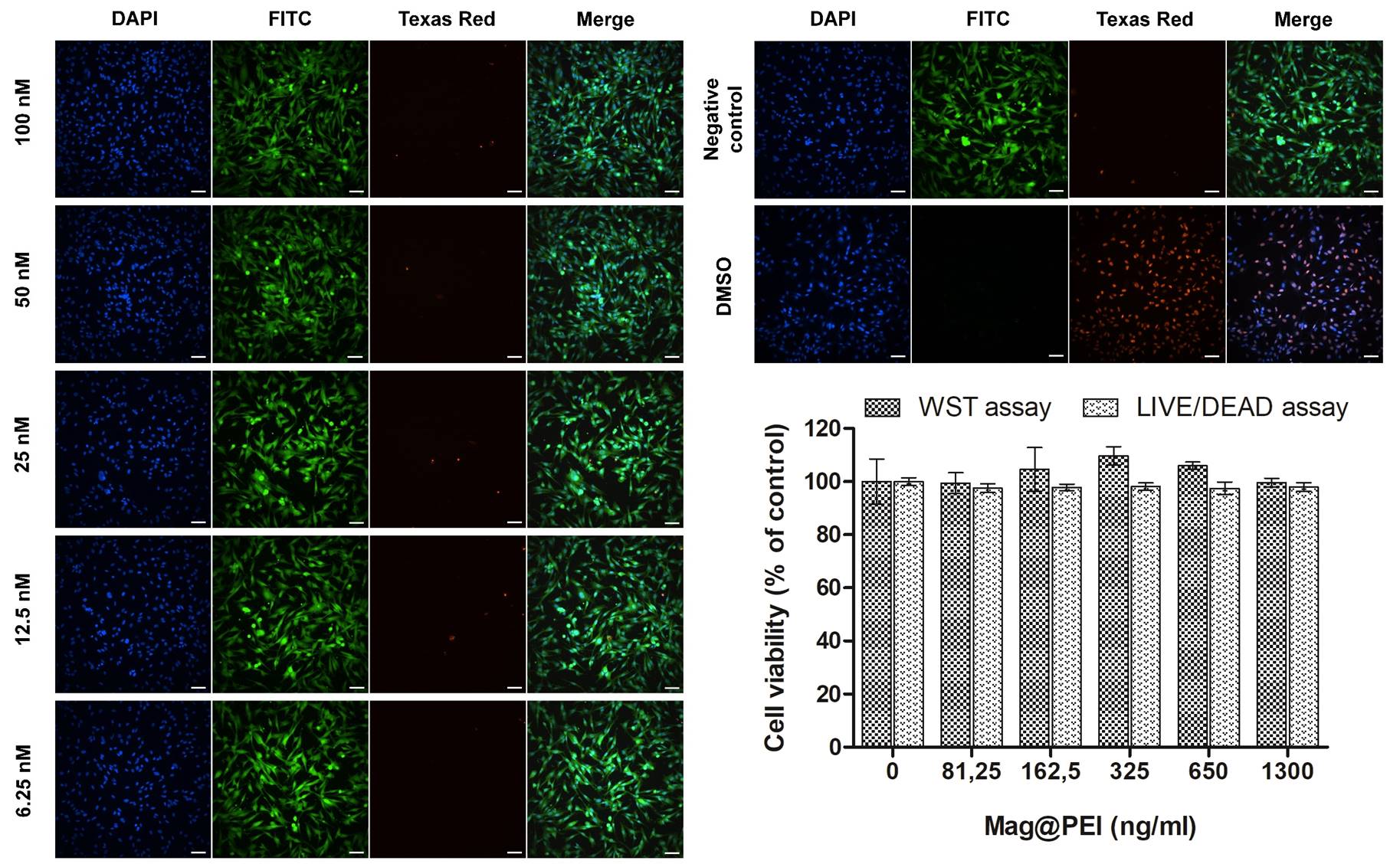


Figure B. Representative high-content images of U-118 cells exposed to Mag@PEI nanoparticles at various concentrations. 10% DMSO was used as a positive control. Images were obtained using different filters to detect nuclei (DAPI), live cells (FITC), and dead cells (TexasRed). The scale bars denote 100 µm. Cell viability of U118 cells exposed to Mag@PEI.

**SRB assay**

The protein-staining sulforhodamine B (SRB, Sigma–Aldrich) microculture colorimetric assay, developed by the National Cancer Institute (USA) for in vitro antitumor screening was used in this study, to estimate the cell number by providing a sensitive index of total cellular protein content, being linear to cell density. The monolayer cell culture was trypsinized and counted. To each well of the 96-well plate, 100 µL of the diluted cell suspension (5,000 and 10,000 cells) was added. After 24 hours, when a partial monolayer was formed, 100μl of fresh medium with different Mag@PEI concentrations (81.25, 162.5, 325, 650 and 1300 ng/mL) were added to the wells. The cells were exposed to compounds for 24 h at 37°C in a humidified atmosphere (90% RH) containing 5% CO_2_. After that, 100 μL of 10% trichloroacetic acid was added to the wells and the plates were incubated for 1 h at 4°C. The plates were then washed out with the distilled water to remove traces of medium and next dried by the air. The air-dried plates were stained with 100 μL of 0.057% sulforhodamine B (prepared in 1% acetic acid) and kept for 30 min at room temperature. The unbound dye was removed by washing five times with 1% acetic acid and then the plates were air-dried overnight. The protein-bound dye was dissolved in 200 μL of 10 mM unbuffered Tris base (pH 10.5) for optical density determination at 510 nm. All experiments were performed in triplicates. Cell survival was measured as the percentage absorbance compared to the control (non-treated cells).


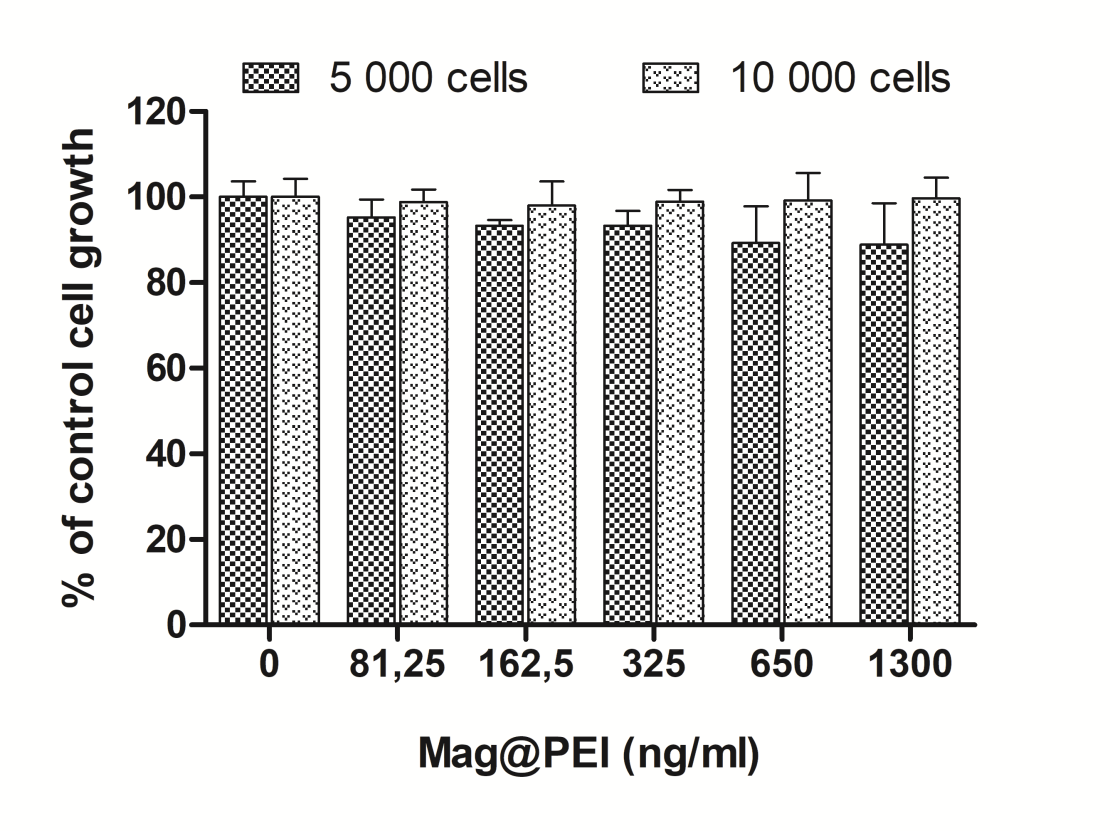


Figure C. Cytotoxicity SRB of Mag@PEI for various nanoparticles concentration.


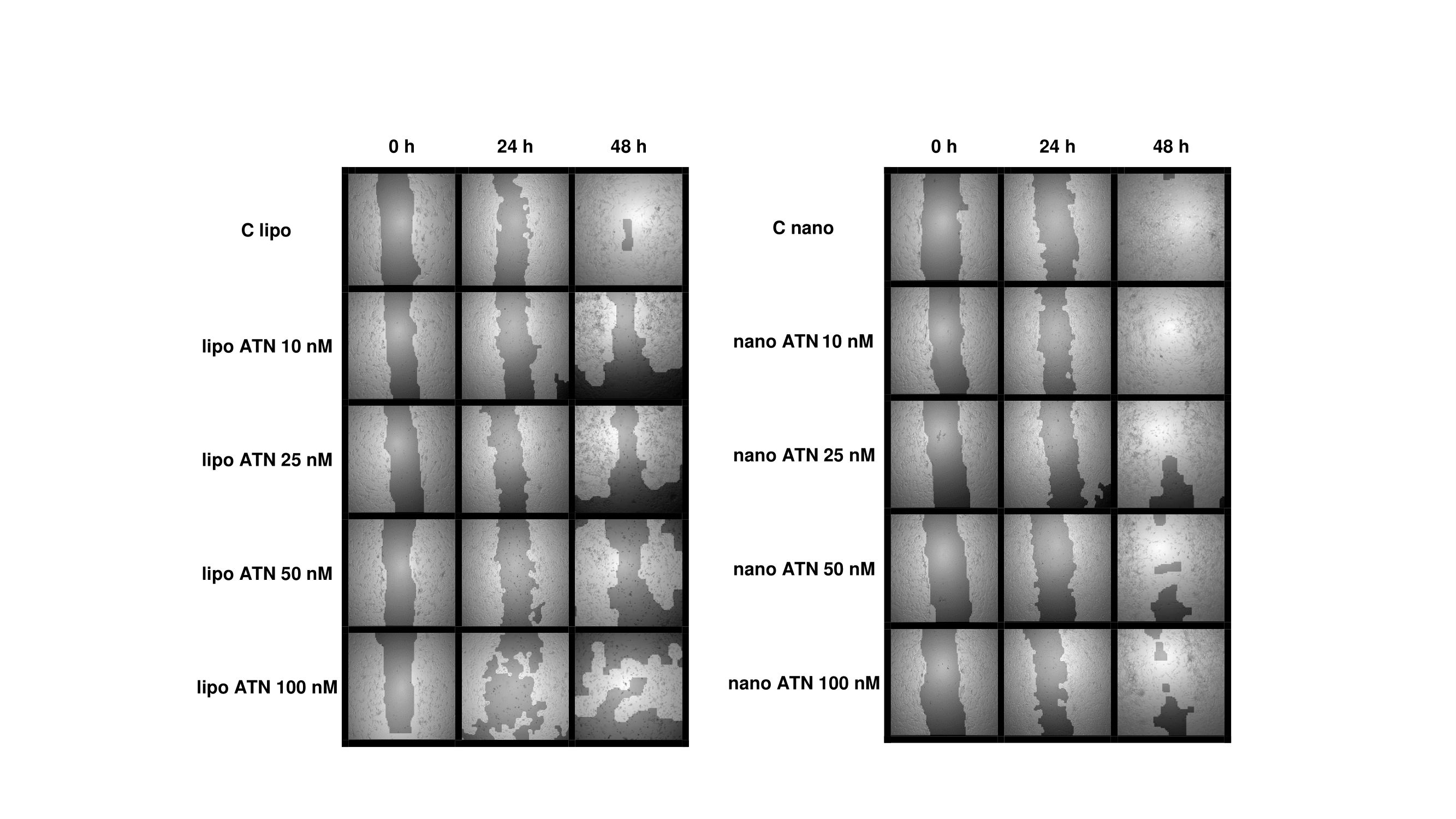


Figure D. Schratch test
